# “We need to confirm at least from two or three”: Healthcare workers’ discretion as gatekeepers in the context of the Ethiopian abortion law

**DOI:** 10.1186/s12939-024-02203-6

**Published:** 2024-06-21

**Authors:** Nega Jibat, Getnet Tadele, Haldis Haukanes, Astrid Blystad

**Affiliations:** 1 Department of Sociology, Jimma University, Addis Ababa University, Jimma, Ethiopia; 2 Department of Sociology, Addis Ababa University, Addis Ababa, Ethiopia; 3 Department of Health Promotion and Development, University of Bergen, Bergen, Norway; 4 Department of Global Public Health and Primary Care, University of Bergen, Bergen, Norway

**Keywords:** Abortion, Abortion law, Healthcare worker discretion, Limited access, Rural Ethiopia

## Abstract

**Introduction:**

Women’s access to legal and safe abortion is a vital means to reduce unsafe abortion, which in turn is known to reduce maternal morbidity and mortality. In 2005, Ethiopia enacted a relatively permissive abortion legislation. However, there is evidence that access to abortion care services may be challenging and controversial even if progressive abortion laws are in place. This article examines women’s access to abortion services from the perspective of healthcare workers in a rural setting in Ethiopia. Drawing on Lipsky’s theory of street-level bureaucrats, the article discusses healthcare workers’ discretion and the substantial authority they hold as gatekeepers to safe abortion services.

**Methods:**

The study draws upon a qualitative, interpretative methodological approach, with in-depth semi-structured interviews with healthcare workers as the key method of data generation. The data was analyzed and interpreted thematically. Healthcare workers’ perspectives were examined with reference to the national abortion legislation and guidelines.

**Results:**

The findings reveal that healthcare workers make decisions on behalf of the women who seek abortion, and they involve parents and partners in abortion-related decision-making processes. Moreover, they assess the social context of the pregnancy such as the marital and economic statuses of the abortion-seeking women in ways that restrict women’s access to legally-endorsed abortion services.

**Conclusions:**

Healthcare workers’ practices in this rural area were found to challenge the basic provisions laid out in Ethiopia’s abortion legislation. Their negative discretion of the legislation contributes to the substantial barriers Ethiopian abortion-seeking women face in gaining access to legal abortion services, despite the presence of a progressive legal framework and guidelines.

## Introduction

Women’s access to legal and safe abortion is recommended as a vital means to reduce unsafe abortion, maternal morbidity and mortality [1–5]. The number of women who die due to unsafe abortion has decreased in many countries after the legalization or liberalization of national abortion laws [6, 7]. It is argued that universal access to safe abortion is the best way to avoid the complications of unsafe abortions, and as the legal grounds for abortion expand, the number of deaths from unsafe abortion decreases. Accordingly, countries with the fewest deaths from unsafe abortion are those which permit abortion on request without or with very few restrictions.

On other hand, although it is established that policy and legal frameworks are important instruments to ensure the right to abortion services, access to legal abortion care is not guaranteed by a mere presence of permissive policies and laws. Reducing unsafe abortion and its consequences is mediated by social, economic, and political factors that influence implementation of the laws [1, 7–9].

The Ethiopian Abortion Law [4] in general and the Technical and Procedural Guidelines for Safe Abortion Care Services in Ethiopia in particular [10], have ensured progressive provisions regarding access to safe abortion care. Although seeking abortion nominally remains illegal in Ethiopia, the Revised Criminal Code [4] provides a more liberal approach to abortion than the law preceding it did. The present abortion law has removed the severe restrictions of the 1957 Criminal Code to access abortion and paved ways for a more rights-oriented abortion care. The law and the guidelines have extended legal grounds for abortion-seeking women to access abortion services (Article 551 sub-articles 1 A-1D, p.356). According to the Procedural and Technical Guidelines of Safe Abortion Care Services in Ethiopia [10, 11], all health centers are supposed to provide first-trimester abortion and post-abortion care, but refer women who seek second-trimester abortion care to hospitals. According to Article 551 (p.355–356), termination of pregnancy by a recognized medical institution within the period permitted by the profession is not punishable in Ethiopia when:

- the pregnancy is a result of rape or incest; or
- the continuation of the pregnancy endangers the life of the mother or the child or the health of the mother or where the birth of the child is a risk to the life or health of the mother; or
- the fetus has an incurable and serious deformity; or
- the pregnant woman is physically or mentally unfit to bring up the child, owing to a physical or mental deficiency she suffers from or her minority status.

An important additional condition stated in the law (Article 551 sub-article 1 A p. 8) is that abortion-seeking women are not required to justify or verify the reason for seeking abortion – a mere statement based on the four legal grounds for abortion is sufficient for gaining access to the service. In the guidelines it is specified as follows:

*Termination of pregnancy shall be carried out based on the request and the disclosure of the woman that the pregnancy is the result of rape or incest. This fact will be noted in the medical record of the woman. Women who request termination of pregnancy after rape and incest are not required to submit evidence of rape and incest and/or identify the offender in order to obtain an abortion service* (Section IV, No 1, p.9).

Accordingly, abortion-seeking women and healthcare providers are to make an informed decision on abortion care with no pressure and interference from a third party. In the pre-abortion care counselling, accurate and sufficient information needs to be provided with regard to the methods of pregnancy termination, including advantages, disadvantages and comparative risks of both continuing the pregnancy and terminating it.

The policy shift on abortion in Ethiopia took place in response to the global agenda of maternal mortality reduction. The law and the guidelines were influenced by the Millenium Development Goals (MDGs) and the 1994 ICPD, which both implied an ideological shift towards viewing people as agents with “reproductive rights”, rather than objects whose fertility is subject to external control. The policy shift was also influenced by the strong desire of the Ethiopian Government to reduce the country’s high maternal mortality rate. This occurred in a context where the sociocultural and religious environments continue to be strongly opposed to a more progressive abortion law [11, 12]. Many studies [1, 8, 9, 11, 12] found that the implementing organizations adopted silence as a strategy in the implementation of the law. This was done to avoid potential public outrage. Tadele et al., analyzing strategies and dilemmas in realizing the new abortion law in Ethiopia, found that the strategy of silence had its advantages, but that simultaneously it was challenging as it prevented the dissemination of knowledge about the revised law, thereby limiting women’s access to safe and legal abortion services [12].

Most previous studies on the abortion law have been undertaken in urban areas [1, 13–16]. Mclean et al. in a study of abortion service providers in Addis Ababa, Ethiopia, found that healthcare workers’ discretion in deciding who gets access to safe abortion entails considerable ethical dilemmas (15). The dilemmas arise when the professionals interpret and implement the law in a bid to balance between their religious faith and values and their strong professional obligations and concerns for women’s health. Ewunetu et al. found that religious distress and the stigma associated with providing abortion influenced the healthcare workers’ view of the law and abortion practices, and it caused an experience severe frustration and burnout [14]. The same authors in a related article argue that, although Ethiopia’s 2005 abortion law improved access to legal abortion services and women’s freedom to choose, healthcare workers were confronted with new moral dilemmas and challenges [13]. The authors state that “the law appears to have opened a large space for professionals’ individual interpretation and discretion concerning whether criteria for abortion are met or not” (p.1).

Studies from various country contexts similarly demonstrate that healthcare workers find the criteria for legal abortion services frustrating [15, 17]. The use of rape and incest as reasons for seeking abortion is both common and contentious [18]. A study from Brazil showed that healthcare workers were particularly grappling with the provisions or the clauses that provide the word of abortion-seeking women to be taken for granted, which allegedly lead many women to use rape as a “cover” for seeking abortion [19]. Syvertsen, in a study from Jimma town in Ethiopia, argues that healthcare workers complain that some women do not use words that fit with the legal grounds for safe abortion care, which does notmake them eligible for the service [16]. In cases when women do not have reasons recognized by the law but still insist on getting the service, Syvertsen [16] found that professionals nonetheless often provide the service by recording the reason for seeking abortion as ‘*She was raped’*. According to Syversten, claims of rape or incest were found to be the most common reasons that women stated to gain access to the service.

Overall, healthcare workers’ perspectives on abortion laws in rural Ethiopia are not well studied. Exploring how healthcare workers perceive, interpret and implement the abortion law in rural contexts where abortion is often strongly disapproved, is important to understand both the abortion-seeking women’s actual access to the services at the grass-roots level and the potential implications of the findings for policy [13, 20]. Most of the research on the topic has been conducted in Addis Ababa [1, 13–15], while only a few studies have been conducted in regional cities like Jimma [16]. This paper explores healthcare workers’ perceptions and experiences with the provision of abortion services in a rural context of Ethiopia in a bid to complement previous studies. This study aims to examine how the 2005 abortion law of Ethiopia and the procedural guideline of abortion services are implemented at the grass root level. Our focus is on the perspectives of the health care providers: their understandings of the law and guidelines and their narratives of clinical encounters with abortion seeking women. The study fills an evidence gap on rural women’s access to abortion care in the context of a relatively permissive abortion law in Ethiopia.

## The study setting

This study was conducted in a rural setting of Ethiopia, in the Oromia National Regional State among the Cushitic-speaking Oromo people residing in Nadhi Gibe district. The district is located in the southwestern part of the State. There are 25 health posts (health facilities at the lowest administrative unit called *kebele*), and five health centers. In principle, a health center is mandated to provide selected types of contraception and first-trimester abortion services. Health posts mainly provide health education and promotion at the community and household levels with special emphasis on women and children.

Family planning (FP) and reproductive health is also one of the major components of the Health Extension Program [21]. Health Extension Workers (HEWs), who are trained for at least one year, are assigned to work at health posts located at the *kebele.* Hence, they work very closely with the community and get to know the care seekers’ families well. As HEWs have limited clinical training, they do not treat patients. Rather, they largely engage in health-related prevention and promotion activities. They are not permitted to provide abortion care as per the Technical and Procedural Abortion Guidelines for Safe Abortion in Ethiopia [10], but they can provide education on reproductive health issues, including on family planning and information about the legal provisions for safe abortion or creating demand for the service. Health centers are staffed with nurses and public health officers who hold a BSc degree.

## Methods

### Study approach and data collection methods

An exploratory and interpretative approach is the basis for this qualitative inquiry. The methods of the study include ethnographic observation, in-depth interviews and review of secondary sources, like abortion-related legal and policy documents. The primary data were collected from district health office leaders, maternal and child health coordinators, healthcare providers from five health centers and HEWs at health posts. Four rounds of two to three months of extended ethnographic observation was undertaken from 2017 to 2019, and a total of 24 interviews were conducted, which include 12 healthcare providers, five MCH program coordinators, two district health office leaders, and five HEWs. Interview guides and observation checklists were employed flexibly during the data collection. The interview guides were prepared in English and translated into Afan Oromo. All interviews were conducted at the workplaces of the healthcare workers (wards and offices) and each took an average of one hour. The first author conducted the interviews, and two research assistants facilitated the sessions. While carrying out the interviews at health service facility settings (health centers and health posts), patients, healthcare providers and health extension workers were observed without delving into true clinical encounters in order to ensure abortion-seeking women’s privacy. The ethnographic observation of the general environment of the clinic was nonetheless important for the in-depth understanding of the study context.

### Data analysis

The interviews were audio-recorded, transcribed verbatim, and translated into English. Thematic analysis was used, following the principles of open, axial and selective coding. The thematic analysis was supplemented by content analysis in the interpretation of the secondary materials, including the provisions of the abortion law and the national guidelines for abortion-related care. The research questions and emerging patterns surrounding healthcare workers’ practices in relation to abortion-seeking women guided the theme formation.

This article employs Lipsky’s work on Street-Level Bureaucrats as an analytical framework to interpret the study findings [22–24]. Lipsky examines a segment of employees in governmental systems which interacts with citizens in the regular course of their jobs. These administrators often have significant independence in making decisions in their jobs, and potentially have extensive impact on the lives of the clients they serve [22]. This segment of workers makes up a category of employees who monitor and implement policies that have been established at higher bureaucratic levels [22, 24–26].

Tummers and Bekkers note that discretions exercised at this level have significance for the effectiveness and legitimacy of public policies [27]. For example, these medium-level bureaucrats can make adjustments to general public policy to fit the specific contexts and needs of people at the grass-roots level. In so doing, the policy emerges as more meaningful to the clients, an effect they refer to as *client meaningfulness.* Alden importantly discusses more problematic or even illegitimate or unlawful discretion and its potential negative effects on the service users [28]. To Alden, the use of illegitimate discretion can potentially lead to detrimental outcomes for service users. The concept of street-level bureaucracy seems highly applicable to the healthcare sector and its workers [22–24, 27], and Alden’s use of the concept of ‘negative discretion’ emerges as particularly useful to interpret the findings of the present material [28]. As healthcare workers seem to employ illegitimate discretion in their interaction with abortion-seeking women or girls, our research draws on the concept to assess healthcare workers’ discretion and its effects on women’s chances to access safe abortion in the context of this study.

### Research ethics

All study participants interviewed were informed about the objectives of the study and potential risks of participating in the study, and questions about willingness to participate in the study were sought through oral consent. A support letter from the district’s health office allowed us to visit the health facilities and conduct interviews with healthcare workers. Observations were done through “hanging around” the health facilities and were conducted with the intention of learning about the study setting, not to engage with or gain knowledge about individuals. Informed consent was therefore not sought from people passing by the clinic, only from those interviewed.

No personal names or other identifiers were used during the data collection or in the analysis and the presentation of the material. Confidentiality and anonymity of the evidence were ensured both during and after the data gathering. Ethical approval was secured from the Oromia Region Health Bureau and the Norwegian Centre for Research Data (SIKT).

## Results

This section will present the results of the study organized by themes. The findings reveal that healthcare workers often use discretion in their interactions with abortion-seeking women in a manner that impedes women’s access to abortion care. However, we also find a few examples of healthcare workers who use discretion to meet the needs of abortion-seeking women.

### Healthcare workers’ use of negative discretion

The identified negative discretionary performance of healthcare providers will be presented in the following three sub-sections: (1) making a decision on behalf of the abortion-seeking women, (2) involving third parties in making decisions about the abortion-seeking women, and (3) using additional / alternative criteria that are not part of the law/guidelines as preconditions for accessing abortion service. Negative discretion generally implies that the providers are not implementing the law as intended.

#### Making decisions on behalf of the abortion-seeking women

During the course of the fieldwork, it became clear that many healthcare providers make decisions on behalf of the abortion-seeking girls/women and challenge them to take a different stance and to change their minds, rather than giving them balanced and legal- and policy-based pre-abortion counseling. A midwife who complained to have been forced to provide abortion care, tried to convince her clients to keep the pregnancy to full term unless the clients insisted on getting an abortion. She asserted her view saying:

*Giving abortion care is a very difficult task. I got into abortion care unknowingly. It was against my personal interest. Hence, before giving them the abortion care services, I try to convince abortion-care-seeking women to keep their pregnancy. I advise and encourage them to avoid seeking abortion. I don’t rush to give abortion care; rather, I focus on educating them not to abort, and I also counsel them about the disadvantages of abortion. As long as a pregnant mother has no serious health problem, we recommend that she should keep the pregnancy instead of aborting it. That is, we mainly try to counsel pregnant women not to abort. This is all we do with abortion care. So abortion care is the last resort. When a woman totally refuses to carry her baby to the full term, I give abortion care.*

The quote shows that the healthcare worker is more preoccupied with making the woman change her mind than establishing the legal reason for the abortion. A HEW similarly reported her proxy decision making on behalf of an abortion-seeking woman as follows: “… *For instance, a woman came to me and asked me for abortion care services. However, I told her to give birth because she was married.”* Another HEW said, “*I told her* [the abortion-seeking woman] *it was difficult to abort a six-month pregnancy, provided her with counseling services, and accompanied her to her home*.”

According to the abortion care procedural guidelines, this latter HEW should have referred the case to a health center, but she did not do this as she believed that being married and having moved far into the pregnancy were against the acceptable norms in terms of gaining access to abortion services. Her decision on behalf of the pregnant woman thus contradicted the norms of the service provision, which tells healthcare workers to counsel abortion-seeking women by explaining both the advantages and disadvantages of abortion and encourage them to seek abortion based on legal grounds (Article 551 sub-articles 1 A-1D, p.356). Most importantly, when legal requirements are met the choice should be left to the abortion-seeking woman. The healthcare providers are then expected to provide abortion care or refer the woman to a place where she can obtain the service. However, staff in health centers which do not provide abortion care may not give the women proper advise. As a result, the abortion-seeking women are left in circumstances where they find it difficult to access the safe abortion services. For instance, a midwife working at a health center that did not provide abortion services said, “*We tell them the disadvantages of undergoing abortion. Then, they may go to other districts for abortion care, but I don’t know much about that.”*

#### Involving third party: endangered anonymity

The findings revealed that in their encounters with abortion-seeking women, healthcare workers involve third parties like parents, partners, local leaders, health extension workers, friends and even the rapist in the decision making regarding abortion care. For instance, a midwife said, *“I first consulted her mother; I also informed the father*.” They were found to commonly require abortion-seeking women to bring any of these third parties to justify the abortion and endorse the abortion-seeking women’s decision. A story shared by a HEW is a typical example that reveals the involvement of third parties in the decision-making process. She narrates the story as follows:

*One day, a six-month-pregnant young girl came to my office seeking abortion care. I first consulted her mother and told her that premarital pregnancy could happen among girls. I informed her that her daughter got pregnant and advised her that once the pregnancy occurred, her daughter should not be exposed to different health risks. In the beginning, the mother was shocked. However, gradually I persuaded her, and she accepted my advice. In consultation with the mother, I also informed the father. Like the mother, the father was shocked in the beginning. Nevertheless, I helped him calm down and control his emotions. In this way, I resolved the problem. As a result, the young woman adjusted herself to the situation, and her parents provided her with the necessary support. I also continuously visited and supported her at home or offered her to visit me at the health post any time. When the pregnancy was due, I took her to the health center for the delivery service and she safely delivered there. After recovery, her parents took her back home. After staying with her family for some time, she went to Finfinnee (*Addis Ababa*). She dropped out of school due to the unwanted pregnancy and birth.*

The story demonstrates that the measures taken by the HEW are not in line with the abortion law and abortion care procedural guidelines. The guidelines stipulate that an abortion-seeking woman should be referred to a health center (for potential further referral). Referring the case directly to a hospital, if that is practically more appropriate, is also possible, as observed during the data collection. In fact, due to geographical proximity and accessibility, sending clients directly to hospitals may at times be easier and more useful than referring women to the health center of the catchment area, which may or may not offer abortion services. The story also involves elements of the proxy decision making discussed above. In this case, instead of providing the abortion seeker with the counselling service about her options, her parents were consulted without the consent of the girl.

Healthcare workers’ decision to involve the abortion seekers’ partners in the decision making demands particular attention, as this was commonly encountered. In the process of making decisions about the fate of the pregnancy, it was found that healthcare workers would involve the partner without the consent of abortion-seeking women. This practice contrasts with the principle of ensuring the abortion-seeking woman’s privacy, and the fact that the abortion seeker is the primary decision maker about whether or not she wants to keep the pregnancy. In such cases, the anonymity of the abortion-seeking woman is not kept, as is the case in the following example:

*If two partners disagree on whether abortion should be sought, we involve the HEWs and the ‘kebele’ manager who go to the partners’ home to resolve the issue through negotiation. If they fail to agree, we take the case to religious leaders. If the husband still refuses to accept the religious leaders’ decisions, we have nothing else to do but apply the abortion law of the country. That is, as the law supports us, we administer abortion care for the woman who seeks it* (Healthcare provider at a health center).

Another healthcare worker had a similar stand on the importance of consulting and getting confirmation from a third party:

*We need to confirm at least from two or three of the young woman’s friends. It is then that we should help. I know a case around Gibe where a family went to visit relatives, leaving at home a young woman and a young man who had blood relations. The young man and the young woman were sleeping together, sharing a bed, whereby the young man raped the girl. After some time, she came and told us that her menstrual cycle had stopped. When she was tested for pregnancy, the result was positive. She told us that her uncle raped her and that she was going to take the Grade 8 national examination. Then, we asked her to call the man, but when he heard this, he disappeared, dropping out of school. When we asked her to bring someone who could confirm this, she told us to call her mother. Her mother came and her father was also informed. After confirming the cause, we referred the case to Sokoru health center in an adjacent district where the fetus was aborted in a private clinic.*

Providing abortion care without the consent of the husband indeed seems to be an exception in the study area. Of note is a midwife’s assertion:

*But sometimes, when a married woman complains that her husband is not providing the necessary care and support for his children, we give abortion care after she signs the abortion agreement form without the consent of her husband. However, as much as possible, we urge couples to reach an agreement on abortion care. If not, it could lead to a conflict between the couples, and may even cause divorce.*

#### Using extra-legal criteria as preconditions for abortion

In addition to healthcare workers’ discretion discussed in the preceding sections, we also found that some healthcare workers use additional, but not legally endorsed, criteria to either provide or deny access to safe abortion care services to girls and women. Such criteria included, for example, using incorrect time reference for the pregnancy, checking the marital status of abortion-seeking women, verification of reasons for seeking abortion, and making family planning a precondition for providing the abortion service as discussed below.

#### Misconceptions about timing of pregnancy and seeking abortion

The following quote reveals that some HEWs would refrain from referring abortion-seeking women to hospitals or clinics based on the timing of their pregnancy, albeit not in accordance with the timing established in the abortion care guidelines. A HEW stated:

*We have been told as part of our health education that if a mother aborts a fetus that is more than three months, it causes serious health damage to the mother. Since it is highly risky for the life of the mother, we do not encourage undergoing abortion after three months of pregnancy. It is better for her to give birth to the child. I think, if the fetus is less than three months old, abortion is acceptable because it does not cause any harm to the mother.*

The quote implies that either the HEW misunderstood the provisions about time limits detailed in the guidelines or was not willing to apply them. It also seems that her assumptions about the consequences of seeking abortion are misleading, given that she states potential harms that are not presented in the guidelines. A similar confusion was observed among other HEWs and some healthcare workers at health centers. For example, as noted across many interviews, there was a strong tendency of rejecting second trimester abortions under the pretext “*It is difficult*”. As a result, referrals of second-trimester cases to hospitals were rarely reported by the healthcare workers. The guidelines, however, clearly state that, while first-trimester abortion care should be provided at health centers by relevant health personnel, cases of second-trimester abortions should be referred to a hospital to be safely managed by trained physicians.

#### Marital status as a precondition to access abortion care

Systematic checking of marital status as part of the decision-making process for abortion care is another example of extra-legal demands from the healthcare workers. A healthcare worker asserted, “*We always check whether a woman has a husband before giving abortion care.”* Although attempts to know about the marital status of an abortion-seeking woman is not a problem in itself, forcing the woman to ensure the consent of her husband for seeking abortion care, which happened in cases of abortion-seeking women in the study area, is not in line with the stated provisions. Another healthcare worker, who had experience of quarreling with a husband when he (the healthcare worker) administered a contraceptive to the wife, argued that he would not provide abortion care without the consent of husbands. He explained:

*For married couples, we do not provide abortion care if a woman comes alone or if her husband has not given his consent. Thus, we insist that she should persuade her husband and bring him to the health center.*

***“If a woman refuses family planning, we don’t give abortion care”***

Requiring that an abortion-seeking woman should use contraceptives as a precondition to get access to abortion care was another unofficial requirement encountered. This goes against the principle of the safe abortion guideline that reads: “*Family planning services are based on a free and informed choice and the availability of methods*” (P.8). The abortion care guidelines highly encourage abortion care providers to integrate pregnancy termination with family planning services, including contraception, to prevent unwanted pregnancies in the future. That is, while giving abortion care, healthcare providers are expected to encourage or counsel abortion-seeking women to use contraceptives, but they are not to make contraception use a precondition to access abortion care. However, we found that some healthcare providers made contraceptive use mandatory to access abortion care. One of them said:

*We also give counseling to abortion-seeking women on how to use family planning services to prevent unwanted pregnancy in the future. Thus, we try to convince abortion-seeking women to use the services before giving them abortion care. If an abortion-seeking woman refuses to use family planning, we do not give her abortion care because she will make the same mistake in the future.*

This quote again demonstrates healthcare workers’ discretion in giving access to women or preventing them from gaining abortion services, in this case based on contraceptive compliance.

Health providers’ discretion that often worked in disfavor of the abortion seeking women, needs to be understood in a context where healthcare workers operate in the broader normative contexts of the rural communities, where they live their lives not only as health workers, but also as daughters, mothers, wives, aunts etc. The anti-abortion sentiments and norms highly prevalent in the communities is likely to constrain their professional conduct. Knowledge obtained in other arenas of the fieldwork suggests the presence of religious and cultural norms which are highly disapproving of abortion. The fact that only two of the five health centers in the district provide abortion care, and the lack of referrals from the health centers to health facilities that do provide care, moreover speaks to healthcare workers who operate outside of the legal framework. What is more, few of the healthcare providers had received abortion care training that is considered a precondition to provide the service. As the training is not made mandatory for health center staff, many are not willing to attend the training due to their religious and cultural convictions against abortion.

### Healthcare workers’ discretion to meet the women’s needs

This subsection presents practices of healthcare workers who do not use negative discretion but rather interpret the law and the guidelines in a way that helps women access abortion services. Some of the healthcare workers interviewed tend to carefully abide by the law and the guidelines as far as their level of understanding allowed. Good knowledge about the legal provisions is expressed by the following healthcare worker, who said:

*There is a guideline that clearly states the rules of abortion care. According to the guideline, we give abortion care if the fetus is highly dangerous for the mother’s life due to illness or other related health concerns. Also, we give abortion care if the mother cannot care for the child due to health, age, disability or other risk factors that may hamper her capacity.*

There were also other healthcare workers who were willing to give the services to the needy clients and who wanted to interpret the laws in favor of the abortion-seeking women or girls. A healthcare provider at a health center, for example, revealed her readiness to provide the service:

*We give priority to the women’s preference, health and life. We do not require abortion-seeking women to produce evidence for their claims or to prove their marital statuses as long as they mention one of the four criteria for legal abortion. Healthcare provision demands keeping secrets of a patient so that her words are respected, and saving her life is our primary concern. We give abortion care services for a woman who claims she has no husband even if we suspect that she does.*

This healthcare worker interpreted the law in line with its intention; that is, meeting the woman’s health and survival needs while observing professional ethics of retaining the anonymity of the patient. Yet, in the same example, it is simultaneously observed that the healthcare worker wrongly understood marital status as a criterion for seeking abortion. This indicates that healthcare workers may partly abide by the law, and partly use discretion based on their level of understanding of the legal and policy basis. One of the healthcare providers articulated the concern that emanated from such sympathy with the life conditions of the abortion seeker as follows:

*People in this area are very poor and life is very difficult here. It is difficult for families to raise two children, let alone five or six. We see their economic conditions during delivery. We know the food they bring for mothers who give birth here. If we refer abortion-seeking women to other health facilities, they may not be able to go there for economic reasons. In this area, it is very difficult even to get their children treated when they get sick because of the limited resources they have. They are worried even about transportation. If we give the services here at local health facilities, we could do it in an affordable manner. That is, we give her abortion care if we believe that she is unable to raise the child. Furthermore, we give abortion care for girls attending schools in primary or secondary levels. For female students who experience unwanted and teenage pregnancy, we give abortion care and advise her to use contraceptives for the future. If a young woman is less than 18 years old and has no husband, we give her abortion care. We also give abortion care when a lactating mother faces an unintended pregnancy. Furthermore, we give abortion care if pregnancy results from incest, which is morally and socially unacceptable in our community. These are the ways the abortion care guidelines dictate us in giving the care.*

This healthcare worker followed a mix of legal and extra-legal criteria in providing abortion service with the intention of assisting the abortion-seeking women in situations of life not compatible with motherhood. The healthcare worker correctly cites some of the legal grounds of abortion, including incest, being a minor, life and health threats as reasons to access abortion, but she mistakenly or deliberately considers abortion-seeking women being students regardless of age, having many children, and not having a husband as justifications for providing the service.

Another healthcare provider similarly expressed a combination of supportive and legal views related to abortion-seeking women as follows:

*When a woman reports experiencing rape, we check for pregnancy and HIV. If the pregnancy test is positive, we (*health center staff*) refer her to health facilities where she can get the service and contribute money for her expenses of the abortion care services. If the test is negative in both cases, we advise and counsel the woman how to protect herself in the future. We advise her to use different options if such a thing happens again. After the advice, we give her an implant, telling her that the rape and unwanted pregnancy could happen in the future. We also give her condoms if she is willing.*

## Discussion

This article focuses on healthcare workers’ extensive use of discretion in their interaction with abortion-seeking women that we encountered in our material. The discretion is often in conflict with the provisions of the 2005 Abortion Law and the Technical and Procedural Guidelines for Safe Abortion Care Services in Ethiopia [10, 11], as detailed above. In the sections below, we discuss the findings with reference to Lipsky’s street-level bureaucracy as well as in relation to other relevant studies [1, 7, 9, 12–15, 29]. The findings show that the progressive abortion policy and legal frameworks of Ethiopia are often not known, are misunderstood, or are ignored and challenged by the healthcare workers in the study area. We found that healthcare workers’ discretion interpreted and often distorted the abortion law and guidelines as they saw fit. Based on the study findings, we argue that in the study area the abortion regulatory framework is not sufficiently binding and is only partly attended to. This has severe implications for women’s actual access to abortion services and thus for their health and lives, as women who are denied the service are likely to seek unsafe abortion measures. Barriers to learning more about the implementation of the guidelines occur partly due to the lack of funds for continuous training of abortion providers. However, the challenge is also located at a more fundamental level and is embedded in the negative sentiments to abortion in the community. This prevents knowledge about the abortion law and guidelines to be openly spread, even among the healthcare workers who are to implement the policies in their day-to-day clinical work. The strategy of silence adopted by the implementing organizations has as such also prevented the dissemination of knowledge about the abortion law and services [12].

Healthcare workers’ discretion can fruitfully be interpreted and discussed in light of Lipsky’s theory on street-level bureaucracy. Lipsky’s theory is relevant as healthcare providers take the abortion law, and procedural and practical guidelines into their own hands and interpret them as they see fit, including making personal judgments about the women’s eligibility for an abortion. Mclean et al. in their study from Addis Ababa, state that “where the law makes the door slightly open, healthcare workers become important in deciding who gets access to safe services and who doesn’t, thus creating considerable ethical dilemmas” (p. 1) [15]. This study, from a typical rural setting of Ethiopia, reveals that healthcare workers play an even more invasive role in deciding women’s access to safe abortion. As there were few attempts by the healthcare workers to hide the lack of adherence to the guidelines, it is clear that there was a fundamental lack of knowledge about their content. It also suggests that few or no attempts were made to administratively follow up on practices not in line with the stated policy. Non-adherence to the policy thus seemed to have no negative repercussions for the health workers themselves. Rather, they seemed to follow up on the abortion requests in a manner expected of and respected by their communities.

We found that health workers breach women’s confidentiality and involve external individuals, including partners, in the decision making regarding abortions. We also found that healthcare workers show signs of a paternalistic attitude towards their clients and act as proxy decision makers in abortion-related clinical decision making. That is, healthcare workers’ decision on behalf of and against the interests of abortion-seeking women seems to emanate from healthcare workers’ paternalistic thinking, indicating a notion that abortion-seeking women do not have adequate understanding of the negative consequences of seeking abortion. Abortion-seeking women’s limited voice in making decisions about their pregnancy reveals healthcare workers’ discretionary power and women’s limited potential or agency to control their fertility.

In line with previous studies, the findings of this paper indicate that women may have severely limited access to legal abortion care services, which is partly related to the healthcare provider they happen to meet at the health facility. This implies that the existence of enabling policies, laws, and implementation guidelines on abortion care does not guarantee access to the services [1, 7–9]. Indeed, the findings from this study reveal that access to abortion care is largely constrained by care providers’ use of extra-legal discretion in their interaction with abortion-seeking women. This emerges as a prime example of street-level bureaucrats’ engagement in providing their own interpretations of key policy documents to the detriment of the women who were to benefit from them. In many of the examples presented above, the women’s health and futures are thus determined not by the rights entailed in the law and guidelines but by personal assessment of healthcare workers who, either willfully or out of lack of knowledge or misinterpretation, stop them from benefiting from policies and laws.

Many healthcare workers believe that seeking abortion overall is riskier than lack of access to the services. As a result, they use negative discretion against abortion-seeking women as a measure of safeguarding them from dangers of experiencing abortion, even in cases where the quest for abortion is made on legal grounds. A position emerges where many healthcare workers find that abortion should be avoided on almost all grounds, as demonstrated by their intense attempts to advise and encourage women to refrain from seeking abortion. The findings reveal that the encouragement given to women and girls not to seek abortion care often disregards the legal grounds for abortion. This indicates a street-level bureaucratic approach, where the law and guidelines are set aside due to contextual concerns, for example the fear of being confronted by an angry spouse.

Using additional and inappropriate criteria as preconditions for restraining abortion-seeking women’s access to the services seems to be based on two grounds. Either the healthcare workers have not fully grasped the law and the practical procedural guidelines or they systematically challenge the implementation of the abortion care framework and policy based on their personal, social and religious convictions [13–15]. It is important to reflect on the fact that healthcare workers who took part in the study may not have been as open about their discretion if they knew that they were disclosing acts against established law and regulation. In this context, it is vital to consider that the kind of discretion described in this paper takes place among healthcare workers who serve in a cultural and religious setting with strong anti-abortion normative patterns [12]. This context is likely to underlie the dynamics of street-level bureaucratic maneuvering among the healthcare providers.

Alden’s concerns related to having little information about the policy is highly relevant in this context. Vedung and Lipsky contend that street-level bureaucrats deliberately develop mechanisms of discretion to cope with psychological threats and conflicting, ambiguous, contradictory and unattainable role expectations [24, 25]. This point resonates strongly with the interpretation of our study findings; it is likely that the healthcare workers operate under conditions of severe pressure to avoid supporting or performing acts that are perceived by community members as illicit.

The substantial power healthcare workers enjoy and their extensive impact on abortion-seeking women’s lives and futures speak to the immense impact implied in their roles as street-level bureaucrats. However, it is also important to note that there are healthcare workers who do attempt to abide by provisions of the laws and guidelines and/or use their discretionary power in favor of the abortion-seeking women. Yet, those who use positive discretion are not always law-abiding. Their use of positive discretion reveals that they strive to make adjustments to the policy provisions to fit the rural context and the needs of abortion-seeking women, in line with what was found in the study conducted by Tummers and Bekkers [27] .

## Conclusion

The findings of this study indicate that there are substantial barriers to Ethiopian women’s possibility of gaining access to legal abortion services, despite the presence of a progressive legal framework and operational guidelines. According to the legal provisions, abortion care providers are expected to accept women’s reasons for seeking abortion at face value as far as the claims fall within the legal grounds to access abortion care. Our study findings demonstrate that a few healthcare workers use positive discretion to meet the needs of abortion-seeking women. However, it is evident that healthcare workers commonly use their power of discretion to prevent women from having abortion, either out of lack of knowledge or misunderstanding of the established legal and policy frames, or knowingly, with the purpose of preventing legal abortions from being conducted in the context of strong anti-abortion norms. The discretion clearly poses challenges to the aims of the progressive legal grounds for abortion in Ethiopia. Our findings alert us to the fact that a well-intended permissive abortion policy will have limited or no impact on women’s, access to safe abortion services if its content is not known and properly enforced or adhered to by health care providers. Making sure that healthcare workers learn about the abortion law and guidelines, as well as the potential consequences of unsafe abortion on women seeking the service, is crucial to improve the situation. A prime recommendation is thus to ensure continuous training of abortion providers in the content of the policy and enforcing its proper implementation. This seems particularly urgent in rural areas of the country where healthcare workers may be particularly prone to be embedded in and influenced by community norms and expectations.

Given that the legal frameworks are national and that the powerful culturally/religiously-embedded anti-abortion norms and sentiments are found throughout Ethiopia, it is likely that the findings of the present study are relevant also to other settings in rural or even in urban Ethiopia. Even though anonymity concerns limited us from directly observing the provider-client interactions in abortion clinics and obtaining abortion-seeking women’s first-hand experiences, we believe that this study has significance in addressing knowledge gaps about access to abortion in the rural context of Ethiopia.

## Data Availability

No datasets were generated or analysed during the current study.

## Abbreviations

HEW: Health Extension Worker
MDGs: Millennium Development Goals
ICPD: International Conference for Population and Development
MCH: Maternal and Child Health
FP: Family Planning
